# Clinical characteristics of behçet’s disease in palestine, a retrospective cohort study

**DOI:** 10.1186/s41927-025-00544-5

**Published:** 2025-07-24

**Authors:** Abdalrahim Daraghma, Lamar Baidoun, Samaa Nazzal, Moath Hattab, Basil Jalamneh, Mahdi Abusalameh, Refat Hanbali, Qusay Abdoh

**Affiliations:** 1 Department of Medicine, Faculty of Medicine and Allied Medical Sciences, An-Najah National University, Nablus, Palestine; 2 Fairview Hospital, Cleveland Clinic Foundation, Cleveland, OH USA; 3 Mediclinic Airport Road Hospital, Abu Dhabi, UAE; 4 Department of Rheumatology, An-Najah National University Hospital, Nablus, 44839 Palestine; 5 Department of Gastroenterology, An-Najah National University Hospital, Nablus, 44839 Palestine

**Keywords:** Behcet disease, Vasculitis, BODI, ICBD, Palestine

## Abstract

**Background:**

Behçet’s Disease (BD) is a chronic, systemic vasculitis of unknown etiology that affects multiple organ systems. It is characterized by recurrent oral and genital ulcers, ocular involvement, affecting arteries and veins of all sizes. It is more prevalent in countries along the ancient Silk Road. Diagnosis is primarily clinical, as there are no specific laboratory tests. The International Criteria for BD (ICBD) was developed to improve diagnostic accuracy. Management requires a multidisciplinary approach, with treatment strategies depending on disease severity. Despite BD’s significant morbidity and diverse clinical manifestations, its prevalence and characteristics remain to be described in Palestine. This research provides critical insights into disease patterns and contributes to improved diagnosis and management in Palestine.

**Methods:**

A retrospective cohort study was conducted in the period from Aug 2024 until March 2025 in rheumatology clinics across the West Bank and Jerusalem. 60 Patients diagnosed with BD based on ICBD (score ≥ 4) were included. Exclusion criteria were cognitive impairment, incomplete records, or non-residence in Palestine. Sixty patients were enrolled. Data was collected via chart review and patient interviews. Disease-related complications were assessed using the Behçet’s Overall Damage Index (BODI) to ensure standardized evaluation.

**Results:**

The male to female ratio was 1.14:1. In addition, the most common initial clinical presentations were oral aphthous ulcers (96.7%), genital aphthous (86.7%), ocular lesions (66.7%), skin lesions noted (46.7%), while vascular lesions occurred in (30%). Neurological manifestations and positive Pathergy test were (25%) and (18.3%), respectively. Regarding complications, the most common were vascular events (36.7%), skin ulcerations (33.3%), mucocutaneous scars (20%), avascular necrosis (13.3%), osteoporotic fractures (10%). Regarding complications in the eye, anterior segment changes presented (15%), posterior segment changes (8.3%), visual impairment in one eye (33.3%), and (13.3%) in both. Neurological complications were less frequent and there was no difference in characteristics between the genders.

**Conclusion:**

The most common manifestations were oral aphthous ulcers, followed by genital ulcers, neurological manifestations, and pathergy was the least frequent. The most frequently reported complications were vascular events, skin ulceration, and visual impairment, while neuropsychiatric complications were the least frequent, and there was no gender difference in BD characteristics.

**Research registry number:**

No trial registry number.

**Supplementary Information:**

The online version contains supplementary material available at 10.1186/s41927-025-00544-5.

## Introduction

Behçet’s Disease (BD) is a chronic auto-inflammatory systemic vasculitis of unknown aetiology, affecting multiple systems. It includes mucocutaneous manifestations, characterized by recurrent oral and genital ulcerations, ocular manifestations, markedly chronic relapsing ocular involvement, and systemic vasculitis involving all sizes of arteries and veins [1]. Although it is not geographically restricted, the ancient Silk Road, which connected the East and West, was the primary location for BD, and the disease is more prevalent in these countries [2]. In addition, some studies suggest a possible relation between the disease and HLA-B51 allele, especially those from Turkey and Asia [3]. BD is a rare disease with different prevalence among many countries, for example disease prevalence is 6.6 per 100.000 for North Jordan [4], 20–420 per 100,000 for Turkey) [5, 6], 20 per 100,000 for Saudi Arabia [7] and 7.6 per 100,000 for Egypt [8]. The diagnosis of BD is generally based on clinical criteria, as no specific laboratory tests are available [9, 10]. This reliance on clinical assessment presents notable challenges, emphasizing the need for extensive medical knowledge and experience in recognizing the disease. Although mucocutaneous lesions are the hallmarks of the disease, especially painful recurrent oral and genital ulceration [11, 12], studies revealed that the main presenting symptoms that led to seeking medical care were oral ulcers (30%), genital ulcers (23%), joint lesions (14%) and eye problems (9%) [13]. These variability in clinical manifestations can contribute to delayed diagnosis, leading to the serious potential implications of the disease [14, 15]. Therefore, the International Criteria for BD (ICBD), which considered a highly sensitive and specific set of criteria to diagnose BD, was created from the collaboration of many experts to overcome the diagnostic dilemma and the flaw of the previous criteria [16]. There are different variants of BD with a predilection to affect a certain body system or multisystemic complications [17]. Cardiac complications include coronary or pulmonary arterial aneurysmal rupture [1]. Although it is quite rare, Neuro-Behcet has a central nervous system (CNS) involvement with focal or multifocal parenchymal and non-parenchymal involvement [18]. In addition, ocular involvement including anterior and posterior ocular involvement may lead to permanent blindness [1].

The treatment of BD requires a multidisciplinary approach [19, 20], which depends on the clinical presentation and systems involved. Although mucocutaneous and joint involvements have a fair outcome with colchicine, non-steroidal anti-inflammatory drugs (NSAIDS) and corticosteroids-based topical treatments, more potent drugs with immunosuppressive agents and newer biological therapies are justified for severe manifestations, including visual, vascular, neurological and gastrointestinal involvements [21, 22]. BD has no cure and is associated with significant morbidity and mortality [1]. However, sustained remission which is defined as being in remission for at least six months, is still achievable. In addition, several factors have been reported as predictors of remission, including adherence to therapy, treatment for more than six years, and remission induction in the first two years of the treatment. In contrast, poor outcome was observed in 31.8% of patients and is associated with male sex, younger age of onset, obesity, and having severe disease [23].

BD is characterized by a wide range of variability influenced by different demographic factors such as gender, age, marital status, education status, geographic groups, and the severity leading to variant presentations, variables, responses to treatment, and unpredictable outcomes [24].

Despite BD being an autoimmune vasculitis with diverse clinical manifestations, no studies have been conducted to explore its prevalence and clinical characteristics in Palestine. This lack of regional data limits healthcare providers’ ability to recognize, diagnose and manage BD effectively. By investigating the demographic distribution, clinical features, complications, and prognostic factors, we will provide a much-needed baseline for future research and clinical decision-making.

This study aims to study the demographic, first clinical characteristics reported and its complications. It also aims to assess the distribution of BD among different demographic groups including age, gender, marital status, education level, employment status and smoking habits. Furthermore, it investigates the frequency and severity of BD complications including vascular events, mucocutaneous scars, osteoporotic fractures, ocular impairment, neurological disorders and psychiatric disturbances. Moreover, the study aims to compare BD clinical characteristics and complications between genders to determine if there are any differences, which will give invaluable insights about the different distribution of BD clinical characteristics in Palestine.

In theory Palestine is likely to have a relatively high incidence of BD due to its geographic location along the historic Silk Road, as other countries along this line have a relatively higher incidence of BD, but due to limitation in medical personnel, especially in the field of rheumatology, and the rarity of the disease, leading to very low clinical suspicion of BD by primary care physicians, and the lack of laboratory tests to confirm it when there’s suspicion of BD. Our study seeks to provide important insights into the features of BD in our area, to provide a guide for primary care physicians on when to suspect BD in our population.

## Methodology

### Study design and setting

This retrospective cohort study was conducted across multiple rheumatology clinics, including private and hospital-based clinics, in the West Bank and Jerusalem. However, some prospective elements were included, as additional data were collected via direct patient phone calls to fill in some missing demographic information, and communications were conducted using a standardized questionnaire, reducing the recall bias. Data collection took place between October 2024 and January 2025.

### Study population, inclusion and exclusion criteria

The study aims to investigate the demographic distribution, clinical features, and complications of BD and to assess the distribution of BD among different demographic groups including age, gender, marital status, education level, employment status, and smoking habits. In light of this objective, the study’s target population comprises patients who have been previously diagnosed with BD. We applied inclusion and exclusion criteria to ensure the appropriate selection of the population. Inclusion criteria involve meeting the ICBD, which assigns specific points for ocular lesions, oral and genital ulcers, skin lesions, CNS involvement, vascular manifestations, and pathergy tests. Patients scoring < 3 were considered not to have BD. A score of 3 was considered probable BD, while scores of ≥ 4 were considered sufficient for the diagnosis of BD, and are therefore included [16]. However, patients with mental or cognitive impairment, patients with incomplete medical records, or those who are not primary residents in Palestine were excluded.

### Sample size and sampling techniques

Due to the limited studies on the accurate prevalence of BD and the novelty of this research in Palestine, and the very low number of patients diagnosed with BD, we were forced to limit the sample size to the available number of patients eligible for enrollment. Participants were recruited from various rheumatology clinics, including both private and hospital-based clinics across Palestine. The catchment size for Almakaesd Hospital and the rheumatology clinic from where we recruited our patients has no recent catchment size; however, in 2014, the catchment size for Almakaesd Hospital was 66,000 patients from Jerusalem, the West Bank, and Gaza. The outpatient rheumatology clinics treat approximately 19,000 patients annually. We found 70 patients that met the criteria for BD by using the ICBD, We successfully enrolled 60 patients in the study; the other 10 either declined the study, or we were unable to reach them. Patients who did not have complete demographic information in the medical record were contacted; 13 of them had difficulties in questionnaire filling and were contacted either by a phone call or a face-to-face interview, while Unreachable, 5 patients were excluded. We found 70 patients that met the criteria for BD by using the ICBD, we successfully enrolled 60 patients in the study, the other 10 either declined the study, or we were unable to reach them. Data collected on clinical characteristics included oral and genital ulcers, eye and skin involvement, vascular issues, neurological symptoms, and a positive pathergy test. Complications were assessed using the Behçet’s Syndrome Overall Damage Index (BODI) and included vascular events (such as thrombosis and aneurysms), skin ulcers, mucocutaneous scars, bone issues (osteoporotic fractures, avascular necrosis), eye problems, and neurological or psychiatric conditions. The study also considered demographic and related factors like age, gender, marital status, education, employment, smoking, disease duration, and medication use.

### Study tool and validity

We used ICBD developed in a collaborative study involving 27 countries to improve the diagnostic accuracy for BD diagnosis. It assigns specific points for each domain, ocular lesions, oral aphthosis, and genital aphthosis are each assigned 2 points, while skin lesions, CNS involvement, and vascular manifestations are assigned 1 point for each. When used, the pathergy test was assigned with 1 point. (Table 1) A patient scoring ≥ 4 points is classified as having BD. The ICBD demonstrated a sensitivity of 94.8% and a specificity of 90.5%. This indicates that the ICBD is highly effective in identifying both the presence and absence of BD [16]. We also used BODI which consists of 46 unweighted-items grouped into 9 organ domains, including mucocutaneous, musculoskeletal, ocular, vascular, neuropsychiatry, gastrointestinal, reproductive system with miscellaneous including systemic amyloidosis, malignancy and diabetes, as each manifestation/symptom must have lasted ≥ 6 months to be scored. It demonstrated good to excellent reliability, with a mean Cohen’s k of 0.84 (95% CI 0.78 to 0.90) and a mean intra-class correlation coefficient of 0.88 (95% CI 0.80 to 0.95) [25], in addition, it had good convergent and discriminative validity for the assessment of BD damage [26]. The data, including the ICBD and clinical and demographic characteristics of the patients, were collected by interviewing the patients either on the phone, in person, or by collecting data from their medical records regarding their first presentation to the doctor that led to the diagnosis of BD using a questionnaire (included in supplementary material). In contrast, BD complications were collected using BODI by interviewing the patients for the ongoing complications.

**Table 1:** The scoring system in the international criteria for behçet disease (ICBD)

International criteria for Behçet disease (ICBD) scoring system	
Sign/symptoms	Points
Ocular injuries	2
Genital ulceration	2
Oral ulceration	2
Skin injuries	1
Neurological manifestations	1
Vascular manifestations	1
Pathergy test	1

### Statistical analysis

The data collected from the study were initially coded using Microsoft Excel and imported into the Statistical Package for the Social Sciences (SPSS) version 21 for analysis, using descriptive summary measures for categorical variables, mean and standard deviation for continuous variables. Chi-square and Fisher-exact test were used as appropriate with *p*-value ≤ 0.05 considered statistically significant. Additionally, Relative Risk (RR) with a 95% confidence interval and Cramér’s V test were used to assess potential associations between variables.

## Results

### Demographic characteristics

A total of 60 patients diagnosed with (BD) were enrolled in the study. The sample comprised 32 (53.3%) males and 28 (46.7%) females, with an average age of 36.9 ± 12.8 years. In addition, the majority of participants were married, accounting for 44 (73.3%), while the remaining 16 (26.7%) were single. Education levels showed that 29 (48.3%) had a university degree, 26 (43.3%) had a middle or high school education, and 5 (8.3%) completed primary school. Moreover, employment status was almost evenly distributed, with 31 (51.7%) unemployed and 29 (48.3%) employed. Smoking habits were reported equally as 30 (50%) smokers and non-smokers (Table 2).

**Table 2:** Demographic characteristics for behcet’s disease (BD) patients (*n* = 60)

		*n* (%)
Gender	Male	32 (53.3)
	Female	28 (46.7)
Age (Years)	< 20	5 (8.3)
	20–40	34 (56.7)
	≥ 41	21 (35.0)
Diagnosis since (Years)	≤ 5 Years	33 (55)
	> 5 Years	27 (45)
Age (Years) (Mean ± SD)		36.9 ± 12.8
Age at diagnosis (Years) (Mean ± SD)		30.5 ± 11.2
Marital status	Single	16 (26.7)
	Married	44 (73.3)
Smoker	No	30 (50)
	Yes	30 (50)
Education	Primary school	5 (8.3)
	Middle or high school	26 (43.3)
	University	29 (48.3)
Employment Status	Unemployed	31 (51.7)
	Employed	29 (48.3)
Self-Care	Independent	57 (95)
	Dependant	3 (5)
Compliant To Medications	No	10 (16.7)
	Yes	44 (73.3)
	Maybe	6 (10)
Drugs	Colchicine	43 (75.4)
	Azathioprine	32 (56.1)
	Steroids	30 (52.6)
	Anti-TNF	9 (15.8)
	Cyclosporin A	6 (10.5)
	Methotrexate	4 (7)
	Adalimumab	2 (3.5)

Most participants 44 (73.3%) were compliant with their medications, while 10 (16.7%) were non-compliant. The average age at BD diagnosis was at 30.5 ± 11.2 years, with 33 (55%) diagnosed within five years and 27 (45%) diagnosed more than five years ago. Regarding self-care, the majority were independent 57 (95%) while only 3 (5%) were dependent (Table 2).

Regarding drug prescriptions, 43 (75.4%) were on colchicine, 32 (56.1%) taking azathioprine, 30 (52.6%) on steroids, 9 (15.8%) taking anti-tissue necrosis factor (anti-TNF), 6 (10.5%) on cyclosporin A, 4 (7%) methotrexate and 2 (3.5%) taking adalimumab (Table 2).

### Clinical characteristics and complications of (BD)

Regarding the first clinical manifestation that led to diagnosis, we used the ICBD criteria, which comprise an extensive range of symptoms, including neurological, vascular, and skin diseases [13], minimum and maximum scores were 4 and 10, respectively. Oral aphthous ulcers were the most common, reported in 58 (96.7%) of patients, followed by genital aphthae in 52 (86.7%). Ocular lesions were present in 40 (66.7%) patients. Skin lesions were noted in 30 (50%) patients, while vascular involvement occurred in 18 (30%) patients. Neurological manifestations and positive pathergy test were reported in 15 (25%) and 11 (18.3%), respectively (Table 3).

**Table 3:** Behçet’s disease (BD) characterization and complications distribution

Characterization:	*n* (%)
ICBD (Mean ± SD)	6.2 ± 1.9
Oral aphthous	58 (96.7)
Genital aphthous	52 (86.7)
Ocular lesions	40 (66.7)
Skin lesions	30 (50)
Vascular Lesions	18 (30)
Neurologic Manifestations	15 (25)
Pathergy + ve	11 (18.3)
***Complications***:	
BODI (Mean ± SD)	7.7 ± 3
Vascular Events	22 (36.7)
Skin Ulceration	20 (33.3)
Visual Impairment in one eye	20 (33.3)
Mucocutaneous Scar	12 (20)
Anterior Segment Changes	9 (15)
Avascular necrosis	8 (13.3)
Visual impairment in the second eye	8 (13.3)
MISCELLANEOUS	8 (13.3)
Osteoporotic fracture	6 (10)
Posterior Segment change	5 (8.3)
Peripheral neuropathy	4 (6.7)
Muscle atrophy	3 (5)
Ischemic heart disease	3 (5)
Cerebrovascular accident	3 (5)
Intra cardiac thrombosis	2 (3.3)
Cognitive Impairment	2 (3.3)
Transverse Myelitis	1 (1.7)
Cranial nerve neuropathy	1 (1.7)
Seizures	1 (1.7)
Psychiatric Disturbance	1 (1.7)
Peripheral Neuropathy	1 (1.7)

In contrast, complications associated with BD were also observed. Vascular events, such as thrombosis or ischemic episodes in 22 (36.7%), skin ulcerations were reported in 20 (33.3%), while mucocutaneous scars affected 12 (20%) patients, avascular necrosis was seen in 8 (13.3%) patients, and osteoporotic fractures were present in 6 (10%). Other complications included anterior segment manifestations in the eye, which were presented in 9 (15%) patients, with posterior segment changes documented in 5 (8.3%) patients, in addition, visual impairment in one eye presented in 20 (33.3%) patients and 8 (13.3%) in both. On the other hand, neurological complications were less common, with peripheral neuropathy present in 4 (6.7%) patients and cranial nerve neuropathy, seizures, and transverse myelitis each reported in 1 (1.7%). Moreover, psychiatric disturbances and cognitive impairment were also rare, each occurring in 1 (1.7%). Other complications were noted in 8 (13.3%), including diabetes and malignancy, which were reported by 7 (11.7%) and 1 (1.7%) patients, respectively, though 44 (73.3%) of patients were compliant to medications. In general, BODI scores mean were 7.7 ± 3 with minimum and maximum scores 2 and 15 respectively.

Regarding medications, the most common prescribed drug was colchicine 43 (75.4%) followed by azathioprine 32 (56.1%), steroids 30 (52.6%), Anti-TNF 9 (15.8%), cyclosporin A 6 (10.5%), methotrexate 4 (7%) and adalimumab 2 (3.5%) (Table 3).

We found that five patients (16%) of those using corticosteroids correlated with AVN, and the same percentage for those with osteoporosis. However, other complications were more pronounced. Visual impairment was observed in half of the patients, while skin ulceration was found in 12 patients (40%), mucocutaneous scars in 9 patients (30%), and DVT was recognized in 9 patients also with 5 of them were found to have more than one episode.

While the colchicine usage among patients is at the highest rate, complications correlation is characterized in skin ulceration with 16 (37.3%), DVT and visual impairment share the same percentage with 13 (30%), 10 patients with DVT have more than one episode, and 9 (20%) have mucocutaneous scars.

When it comes to azathioprine, it is as follows skin ulceration conducted in 11 (34.4%), visual impairment in 10 (31.3%), DVT in 8 patents (25%) 6 of them have more than one episode, AVN and mucocutaneous scars were found equally with 5 patients (15%). On the other hand, the rest of the drugs have limited usage.

### Comparing clinical characteristics and complications between genders

#### Clinical characteristics

Oral aphthous ulcers ulcers, a hallmark of BD, were observed in 100% of male and 92.9% of female patients. Genital aphthous ulcers were more frequent in males (93.8%) than in females (78.6%). Ocular lesions were more common in males (75%) than in females (57.1%). Skin lesions were identified in 56.3% of males and 42.9% of females. Vascular involvement was slightly more prevalent in females (32.1%) than in males (28.1%). Neurological manifestations were equally distributed, affecting 25% of both groups. The pathergy test yielded positive results in 18.8% of males and 17.9% of females.

#### Complications

Mucocutaneous scars were more frequent in males (25%) than in females (14.3%). Skin ulcerations were more common in males (43.8%) compared to females (21.4%). Osteoporotic fractures were more prevalent in males (12.5%) than in females (7.1%), and avascular necrosis was observed in 18.8% of males versus 7.1% of females. Ocular complications, including anterior segment changes (12.5% in males vs. 17.9% in females) and posterior segment changes (9.4% in males vs. 7.1% in females), showed no significant gender differences. Visual impairment was more common in males (37.5%) than females (28.6%). Vascular events were more common in females (42.9%) than in males (31.3%). Neurological complications were rare in both groups, and psychiatric disturbances were exclusive to females (3.6%). Miscellaneous complications, such as diabetes and malignancies, were more common in females (21.4%) than in males (6.3%).

In general, comparing clinical characteristics and complications between male and female BD patients revealed no statistically significant differences. The relative risk and Cramér’s V test results indicated weak associations between gender and the studied variables, suggesting that gender does not influence the clinical presentation or complications of BD in this cohort (Table 4).

**Table 4:** Comparison between genders in the different characteristics and complications of behçet’s disease patients

	Male (*n* = 32)	Female (*n* = 28)	Relative Risk (RR)	95% CI	*P*-Value	Cramer’s v
**Characterization**:	n (%)	n (%)				
Genital apotheosis	30 (93.8)	22 (78.6)	1.19	0.96–1.47	0.131	0.223
Ocular lesions	24 (75)	16 (57.1)	1.31	0.89–1.91	0.143	0.189
Oral apotheosis	32 (100)	26 (92.9)	1.07	0.97–1.19	0.214	0.199
Skin lesions	18 (56.3)	12 (42.9)	1.31	0.77–2.22	0.112	0.134
Vascular Lesions	9 (28.1)	9 (32.1)	0.87	0.40–1.89	0.735	0.044
Neurologic Manifestations	8 (25)	7 (25)	1	0.41–2.41	1.000	0.000
Pathergy + ve	6 (18.8)	5 (17.9)	1.05	0.35–3.07	0.925	0.012
**Complications**:						
Mucocutaneous Scar	8 (25)	4 (14.3)	1.75	0.59–5.19	0.301	0.134
Skin Ulceration	14 (43.8)	6 (21.4)	2.04	0.91–4.59	0.067	0.236
Osteoporotic fracture	4 (12.5)	2 (7.1)	1.75	0.35–8.84	0.675	0.089
Muscle atrophy	2 (6.3)	1 (3.6)	1.75	0.17–18.28	1.000	0.061
Avascular necrosis	6 (18.8)	2 (7.1)	2.62	0.58–11.98	0.264	0.170
Anterior Segment Changes	4 (12.5)	5 (17.9)	0.70	0.21–2.35	0.721	0.075
Posterior segment change	3 (9.4)	2 (7.1)	1.31	0.24–7.3	1.000	0.040
Visual Impairment in one eye	12 (37.5)	8 (28.6)	1.31	0.63–2.74	0.585	0.094
Visual impairment in the second eye	4 (12.5)	4 (14.3)	0.87	0.24–3.18	1.000	0.026
Vascular Events	10 (31.3)	12 (42.9)	0.72	7.90–2.78	0.386	0.112
Ischemic heart disease	2 (6.3)	1 (3.6)	1.75	0.17–18.28	1.000	0.061
Intra cardiac thrombosis	2 (6.3)	0	4.39	0.21–87.82	0.494	0.174
Cerebrovascular accident	1 (3.1)	2 (7.1)	0.438	0.04–4.57	0.594	0.092
Transverse Myelitis	1 (3.1)	0	2.63	8.61–16.76	1.000	0.122
Peripheral neuropathy	3 (9.4)	1 (3.6)	2.625	0.29–23.82	0.616	0.116
Cranial nerve neuropathy	1 (3.1)	0	2.63	8.61–16.76	1.000	0.122
Seizures	1 (3.1)	0	2.63	8.61–16.76	1.000	0.122
Psychiatric Disturbance	0	1 (3.6)	0.29	19.56–8.04	0.467	0.139
Peripheral Neuropathy	1 (3.1)	0	2.63	8.61–16.76	1.000	0.122
Cognitive Impairment	2 (6.3)	0	4.39	0.21–87.82	0.494	0.174
MISCELLANEOUS	2 (6.3)	6 (21.4)	0.29	61.36–3.12	1.000	0.218

## Discussion

This study offers a detailed analysis of BD in the Palestinian population, establishing the demographic, clinical, and complication profiles associated with the condition. Inclusion criteria were determined based on the diagnostic criteria suggested by the International Criteria for BD (ICBD) [16]. There are no previous studies investigating the prevalence nor the characteristics of BD in Palestine. As a result, the number of patients available for inclusion was considered to be the sample size. We successfully included 60 patients diagnosed with BD.

We compared the demographics and clinical characteristics in our region with several studies selected to represent different geographical areas from the Middle East, through Europe, and North America. Gender distribution had a very slight male predominance, the male to female was 1.14:1, which is inconsistent with the other countries in the region, as they had a more significant male/female ratio, particularly in regions like the Middle East and Mediterranean Basin, where men were thought to be more affected than women [14, 27]. In addition, recent studies in Saudi Arabia and Egypt suggest that the female ratio was 3.4:1 in both, and 2.4:1 in Jordan [4, 7, 28], whereas in Brazil, Korea, and the United States, females were the predominant [29]. Moreover, young adults between the age of 20 and 40 are the patients typically affected with (BD), although the onset can occur at any age [14, 29], in our study 57% of patients where 20–40 years, 35% were above 41 years and only 8% less than 20 years at the time of diagnosis, which is consistent with other studies.

Although there is no significant difference in age at diagnosis, clinical characterization or possible complication between genders, recurrent painful oral aphthosis (OA), are often the first sign [12] globally, in our study it was seen in 97% of patients which is consistent with the value in other countries in the region, such as Saudi Arabia that had 100% [7], Egypt 87% [28], Turkey 100% [29], Iran 97% [30] these high percentages are consistent with OA being the most common clinical manifestation of BD [31]. On the other hand, genital aphthosis (GA) has the same clinical characteristics of the oral aphthosis, while it appears on the vulva or scrotum and eventually heals with scars [32], it was reported by 87% of patients in our population, consistent with other studies in the region, in Saudi Arabia 87% [7], Egypt 82% [28], Iran 64.6% [30], Turkey 85.6% [29]. Ocular lesion which can involve the retina and uvea. About 10% of individuals have ocular involvement as their initial sign of BD [27], and vascular event 66.7% and 46.7% respectively, while in Saudi Arabia, they were reported as 65% and 57% respectively [7], in Egypt they were 68.7% and 26% [28], in Turkey, they were 47.6% and 10.6 [29]. Moreover, neurologic manifestations including both extra-parenchymal and parenchymal lesions were reported in third of cases worldwide [33]. In our study, it was presented in 25% of cases, while in Saudi Arabia 44% [7], Egypt 25.3% [28], Turkey 2.3% [29], and Iran 3.8% [30]. Pathergy test is a nonspecific hypersensitive skin reaction brought on by a needle prick. This showed up clinically as erythematous induration where the skin injury occurred [26]. This test resulted positive in 18.3% of our patients, while in Turkey 56.1% [29], Iran reported by 52.5% of cases [30], Egypt 28% [28], Saudi Arabia 17.5% [7].

In the Palestinian health institutions, the pathergy test is not basically performed; this may reflect its diagnostic yield. Moreover, physicians reliance on other clinical manifestations and the ICBD criteria may deprioritize its use. This suggests disparities in clinical practice rather than epidemiological differences. Low rates of early diagnosis observed in our study could be attributed to several factors. Limited rheumatology clinics and services in specific areas may delay the suspicion, so the referral to the specialized canter. The prevalence of HLA-B51 may be lower in our local population, which influences the incidence or phenotype. This genetic discrepancy is worth studying in Palestine [34]. 

Family dependence was in 5% of the population and was not directly related to disease itself, where 2 patients were children and the other was 64 years old, bed ridden due to previous trauma resulting in vertebral and tibial fractures. Moreover, BD complications were obtained using BODI, which was designed to identify, describe and measure multi-organ damage in patients with BD [25]. The mean score for all patients was 7.7 ± 3, with minimum and maximum scores of 2 and 15 respectively. In addition, the most reported complications were vascular events (36.7%), which consist of both venous and arterial involvement. This includes complications such as deep vein thrombosis, arterial aneurysms, and other vascular manifestations associated with the disease. The second most reported complication was skin ulceration and visual impairment in one eye, each presenting in (33.3%) of the cases. In contrast, the least reported were neuropsychiatric complications (1.7%), such as transverse myelitis, cranial nerve neuropathy, seizures, psychiatric disturbance, and peripheral neuropathy.

In general, male patients aged 20 to 40 years most commonly presented with oral or genital aphthosis as the first symptom leading to a BD diagnosis. These findings provide valuable insight into the initial manifestations of BD in Palestine, filling a gap in knowledge and helping healthcare providers recognize early signs of the disease more effectively. In contrast, positive pathergy tests and neurological manifestations were the least reported as initial symptoms. This could be due to the limited use of the pathergy test in clinical practice or a genuinely lower prevalence of positive results in this population.

Regarding complications, mucocutaneous damage was the most frequent, followed by ocular damage. These findings emphasize the need for early recognition and proactive management of BD, facilitating prompt therapeutic modifications to minimize complications and enhance patient outcomes.

## Conclusion

Early diagnosis and treatment of BD are the key factors to improving quality of life and reducing complications. However, diagnosing BD remains a significant challenge in daily practice, as it relies on clinical manifestations rather than specific laboratory tests. This makes research on disease manifestations crucial for early diagnosis and increased awareness. In conclusion, the most common manifestations were oral aphthous ulcers, followed by genital ulcers, neurological manifestations, and the pathergy test were the least common. The most reported complications were vascular events, skin ulceration, and visual impairment, while neuropsychiatric complications were the least frequent, and there was no difference in frequency of BD clinical characteristics among genders.

## Strengths and limitations

This study provides valuable insights into BD by analyzing its demographic distribution, clinical manifestations, and complications using the ICBD criteria. It focuses on gender differences and severity patterns, enhances understanding, contributing to improved diagnosis and management in Palestine and the region.

Despite the valuable contributions of this study, it is important to acknowledge its limitations. First, the relatively small sample size (*n* = 60) may not fully represent the broader BD population in Palestine, limiting the generalizability of our results. Additionally, the lack of nationwide epidemiological data on BD prevalence in Palestine meant that our sample was determined by the number of available patients rather than a population-based estimate. Crucially, the retrospective design of the study presents inherent challenges. Reliance on existing medical records and patient-reported histories increases the risk of recall bias and introduces variability in data completeness, potentially limiting the generalizability of the findings to the broader patient population. A retrospective design inherently limits causal inference and may introduce information bias due to reliance on existing medical records. Missing or inconsistently recorded clinical details may particularly affect assessments of disease progression and complications, and may affect the accuracy and comprehensiveness of the dataset.

This study’s retrospective design also poses a potential limitation, as reliance on medical records and patient-reported histories may introduce recall bias and missing data, particularly regarding disease progression and complications that developed post-diagnosis. Lastly, while this study provides an overview of clinical manifestations and complications, it does not assess treatment response or long-term disease outcomes, which are crucial factors for improving patient management. Addressing these limitations in future research will enhance the accuracy and applicability of BD studies in Palestine. To overcome these limitations, future research should consider prospective cohort studies or the establishment of a national BD registry. These approaches would allow for standardized, comprehensive data collection and facilitate long-term follow-up, ultimately enhancing understanding and management of BD in Palestine and similar settings.

## Recommendations

Enhancing awareness and education among general practitioners is crucial for improving BD diagnosis and management. BD can often be misdiagnosed or overlooked, leading to delayed treatment and worsening patient outcomes. For that, we recommend a long-term study tracking BD patient in Palestine would provide valuable insights into disease progression and treatment efficacy. Given the variability in BD manifestations, personalized treatment plans should be explored by studying genetic and environmental factors to create more tailored therapeutic approaches. Additionally, lifestyle interventions should not be overlooked. Encouraging healthy habits such as smoking cessation and stress management may help mitigate symptom severity and enhance overall patient well-being. Finally, maintaining comprehensive medical records for BD patients is crucial. Ensuring that all patient histories, diagnostic criteria, and treatment plans are well-documented will allow future researchers to access valuable data for further studies, improving continuity of care and advancing BD research.

## Electronic supplementary material

Below is the link to the electronic supplementary material.

Supplementary Material 1

## Data Availability

The raw data needs permission from the Palestinian ministry of health to be accessed.
