# Supplementary material for: Clinical characteristics of behçet’s disease in palestine, a retrospective cohort study
Source: BMC Rheumatol. 2025 Jul 24;9:93. doi: 10.1186/s41927-025-00544-5 (PMC12288266; doi:10.1186/s41927-025-00544-5)
Supplement: Supplementary file 1 — Supplementary Material 1 [file 41927_2025_544_MOESM1_ESM.docx]

**Clinical characteristics of Behçet’s disease in Palestine:** **A retrospective cohort study**

Behçet’s disease (BD) is a chronic vasculitis of unknown etiology, which may involve many organs. The clinicians who take care of any chronic disease would like to know the current status of a patient to manage them properly, in this regard the need for developing a standardized assessment of disease activity of BS has been a major concern for clinicians for decades. ICBD is the criteria we have chosen to assess the characterization of the disease in Palestine, which consists of specific points on each one, and later some quotations about the patient's status.

1. Consent form

We appreciate your participation in this survey, which aims to understand the specificity of Behcet's disease in Palestine.

We would like to emphasize that your participation is completely voluntary, and you can withdraw at any time without any impact on the medical services you receive

Consent:

I confirm that I have read and understood the details of this survey.

I know that all information I provide will be completely confidential and will be used only for research purposes.

I understand that my participation is voluntary, and that I have the right to stop participating at any time without any consequences.

By agreeing to participate, I acknowledge that I am providing the information of my own free will.

Agree.

Disagree.

1. patient id:
2. **a total of at least four points for diagnosis of Behçet disease.**

Genital aphthosis

Ocular lesions (anterior uveitis, posterior uveitis, or retinal vasculitis)

Oral aphthosis

Skin lesions (pseudo folliculitis or erythema nodosum)

Vascular lesions (superficial phlebitis, deep vein thrombosis, large vein thrombosis, arterial thrombosis, or aneurysm)

Neurologic manifestations

Pathergy

1. Years Since diagnosis
2. Age
3. Smoking Status

Yes

No

1. Education status

Illiterate or primary school

Middle or high school

University

1. marital status

single

married

1. oral hygiene

good

moderate

bad

1. access to the internet

Yes

No

1. Self-Care or need for someone else for daily tasks

self-care

family

Other:

1. employment status

Yes

No

1. country of birth
2. Medication

*Check all that apply.*

Colchicine

Corticosteroids

Azathioprine

Cyclosporin A

Interferon alpha

Cyclophosphamide

Sulphasalazine

Methotrexate

Anti-TNF

Other:

1. compliant to medication *Mark only one oval.*

Yes

No

Maybe

**(BODI)**

**Behçet's syndrome Overall Damage Index**

1. MUCOCUTANEOUS

Mucocutaneous scar

Skin ulceration

1. MUSCULOSKELETAL

Osteoporotic fracture or vertebral collapse

Muscle atrophy

Avascular necrosis

1. OCULAR

Anterior segment change

Posterior segment change

Visual impairment in one eye

Visual impairment in the second eye

Blindness in one eye

Blindness in the second eye

Cataract

1. VASCULAR

𝐀.𝐃𝐞𝐞𝐩 𝐯𝐞𝐧𝐨𝐮𝐬 𝐭𝐡𝐫𝐨𝐦𝐛𝐨𝐬𝐢𝐬 (𝐃𝐕𝐓)

- - Has more than one episode occurred?
  - Has any episode involved cavae, sovraepatic veins, or cerebral sinus?

𝐁.𝐀𝐫𝐭𝐞𝐫𝐢𝐚𝐥 𝐚𝐧𝐞𝐮𝐫𝐲𝐬𝐦 𝐨𝐫 𝐩𝐬𝐞𝐮𝐝𝐨-𝐚𝐧𝐞𝐮𝐫𝐲𝐬𝐦

- - Has more than one episode occurred?
  - Has any episode involved aorta or pulmonary artery?

Has any episode required vascular surgery?

𝐂.𝐀𝐫𝐭𝐞𝐫𝐢𝐚𝐥 𝐬𝐭𝐞𝐧𝐨𝐬𝐢𝐬 𝐨𝐫 𝐭𝐡𝐫𝐨𝐦𝐛𝐨𝐬𝐢𝐬

Has more than one episode occurred?

Has any episode involved aorta or pulmonary artery?

Has any of these required vascular surgery?

𝐃.𝐌𝐚𝐣𝐨𝐫 𝐭𝐢𝐬𝐬𝐮𝐞 𝐥𝐨𝐬𝐬

1. CARDIOVASCULAR

Constrictive pericarditis

Severe aortic regurgitation

Ischaemic heart disease

Intracardiac thrombosis

1. NEUROPSYCHIATRIC

Cerebrovascular accident

Has more than one episode occurred?

Seizures

Motor or sensory disturbance

Transverse myelitis

Cranial nerve neuropathy

Peripheral neuropathy

Psychiatric disturbance

Cognitive impairment

1. GASTROINTESTINAL

Fistula

Stricture

Perforation

Infarction or resection on any part of GI tract for any reason. Has more than one tract been involved?

1. REPRODUCTIVE SYSTEM

Premature gonadal failure

1. MISCELLANEOUS

Systemic Amyloidosis

Malignancy

Diabetes
